# Beyond metabolism: nutrition, sleep, and psychological wellbeing in children with insulin resistance—a case-control study

**DOI:** 10.3389/fnut.2026.1781309

**Published:** 2026-03-30

**Authors:** Evla Demircioğlu, Merve Pehlivan

**Affiliations:** 1 Department of Nutrition and Dietetics, Institute of Health Sciences, Istanbul Medipol University, Istanbul, Türkiye; 2 Department of Nutrition and Dietetics, Faculty of Health Sciences, Istanbul Medipol University, Istanbul, Türkiye

**Keywords:** Children, depression, insulin resistance, nutritional status, sleep quality

## Abstract

**Objective:**

In this study, it was aimed to compare the nutritional status, sleep quality, and depression levels of children between the ages of 10–18 who were diagnosed with insulin resistance with their healthy peers.

**Methods:**

A total of 60 children, including children diagnosed with insulin resistance (case) and healthy children (control), were included in the study. Children's demographic information, dietary habits, physical activity status, biochemical findings, and two-day dietary intake records were taken, and the Mediterranean Diet Quality Index (KIDMED), Pittsburgh Sleep Quality Index (PSQI), and Children's Depression Inventory (CDI) were applied to the children.

**Results:**

A comparison of the case and control groups revealed that the children in the case group showed significantly higher body weights, BMI means (kg/m^2^), waist circumferences (cm), body fat ratios (%), and BMI z-score according to age compared to the control group (*p* < 0.05). A subsequent analysis revealed that the energy (kcal), carbohydrate (g), protein (g), fat (g), saturated fatty acids (g), polyunsaturated fatty acids (g), and cholesterol (mg) intakes of the children in the case group were statistically significantly higher compared to the children in the control group (*p* < 0.05). The mean KIDMED and Dietary Phytochemical Index (DPI) scores of the children in the control group were significantly higher than those in the case group (*p* < 0.05). There was no statistically significant difference between the groups with respect to the Dietary Inflammatory Index (DII) score (*p* > 0.05). A statistically significant difference in mean PSQI and CDI scores was observed between the case and control groups (*p* < 0.05).

**Conclusion:**

In addition to the nutritional status of children with insulin resistance, it is very important to carefully address, monitor, and improve their sleep quality and depression levels.

## Introduction

Insulin resistance (IR) can be defined as a state of disruption of the normal biological response to endogenous or exogenous insulin hormone or a state in which the amount of insulin hormone required for the normal reaction of the cell, tissue, or organism to occur (quantitatively) is higher than the normal level (1). Many diseases, including obesity, cardiovascular diseases, type 2 diabetes, non-alcoholic fatty liver disease (NAFLD), polycystic ovary syndrome (PCOS), and metabolic syndrome are clinically associated with IR (2).

Risk factors for IR are categorized into two groups: non-modifiable and modifiable. Factors such as genetics, race/ethnicity, and adolescence are among those cannot be changed, while factors such as obesity, eating habits, and physical activity are among the risk factors that can be changed (3).

The most common pathophysiological cause of IR is obesity (4). Childhood obesity is one of the most important public health problems of the 21st century (5). There is a continuous increase in the prevalence of overweight and obesity in children and adolescents due to improper eating habits and lack of exercise (6). WHO reported that 37 million children under the age of 5 and more than 390 million children and adolescents between the ages of 5–19 (160 million obese) were overweight in 2022. Worldwide, adult obesity has more than doubled since 1990, while adolescent obesity has quadrupled (7). Therefore, in parallel with the rising frequency of pediatric obesity, the incidence of IR has also increased (3).

The relatively safe and well-accepted approach to preventing and treating IR is lifestyle changes. Nutritional interventions that emphasize a low-calorie, low-fat diet, one of the lifestyle changes, and increasing physical activity to increase energy expenditure and improve muscle insulin sensitivity are the main approaches in the treatment of IR (2). The Mediterranean diet, which is low in saturated fat and rich in unsaturated fatty acids, vitamins, and other functional components, including various plant-derived bioactive phenolic compounds with antioxidant and anti-inflammatory properties, is associated with greater improvement in IR in obese people than other dietary interventions (8).

Sleep is a biological and behavioral process that is very important for body homeostasis (9). Delayed sleep timing, short sleep duration, and poor sleep quality in adolescents have each been associated with higher IR (10).

Depression is a psychological disorder characterized by persistently low mood, decreased enjoyment, and loss of interest (11). It is reported that there is significant evidence that depression can lead to increased IR and that IR can worsen existing depressive symptoms (12). The study aims to compare the nutritional status (anthropometric measurements, dietary habits, dietary quality, dietary phytochemical indices, and dietary inflammatory indices), sleep quality, and depression levels of children aged 10–18 years who have been diagnosed with IR with their healthy peers.

## Methods

This study was conducted from September 2024 to June 2025. It involved two groups: a case group consisting of children diagnosed with IR between the ages of 10 and 18, and a control group consisting of healthy children. The case group consisted of children who were diagnosed with IR by the doctor who referred them to the nutrition and diet outpatient clinic, and the control group consisted of randomly selected volunteer children studying in secondary and high schools.

The minimum number of samples to be included in the study was set at an effect size of 0.80, a power of 0.95, and a margin of error of 0.05, yielding a minimum of 26 per group using the G^*^Power program (13). Accordingly, a total of 60 children, 30 children diagnosed with IR, and 30 healthy children between the ages of 10–18, were included in the study.

The inclusion criteria of the study were being between the ages of 10 and 18, having been diagnosed with IR in the case group (HOMA-IR >3.16), being healthy in the control group, and not following any diet. Exclusion criteria of the research were taking medication for the treatment of IR, as well as having a chronic disease such as diabetes, cardiovascular diseases, respiratory system disorders, and/or growth retardation. To address potential sources of bias, objective criteria were used to define IR, and all data were collected under standardized conditions by the same trained dietitian (corresponding author). Consistency in measurement and data entry was ensured to minimize measurement and observer bias. The flow diagram illustrating the study selection process is provided in the Supplementary Figure 1.

This research was carried out with the “Ethics Committee Approval” of Istanbul Medipol University Non-Interventional Clinical Research Ethics Committee dated 02.09.2024 and numbered E-10840098-202.3.02-5276. The children who participated in the study and their parents were informed about the research before the study, and the parents of the children were made to sign the “Informed Voluntary Consent Form” indicating that they voluntarily participated in the research.

### Study design

The questionnaire form prepared for the study was applied to the volunteer participants by the researcher using face-to-face interview method. With this questionnaire, demographic information, dietary habits, physical activity status (whether they engage in physical activity and the frequency), biochemical findings, and 2-day dietary intake records of the children were taken. In addition, the “Mediterranean Diet Quality Index (KIDMED)” was used to determine the diet quality of children, the “Pittsburgh Sleep Quality Index (PSQI)” was used to determine their sleep quality, and the “Depression Inventory for Children (CDI)” was used to determine their depression levels.

### Evaluation of anthropometric measurements

Children's body weight (kg) and body fat percentage (%) were measured using the Tanita MC-780 body analyzer (14). Body fat ratios (%) reference curves developed (15) by Kurtoglu et al. (15) for Turkish children and adolescents were used to evaluate the body fat ratios of children. Those with body fat percentage < 10th percentile were evaluated as underweight, those with ≥10th percentile but < 85th percentile as normal, those with ≥85th percentile but < 95th percentile as overweight, and those with ≥95th percentile as obese.

The measurement of the height of the children (cm) was carried out standing, without shoes, with the feet side by side, with the head in the Frankfurt plane (the eye triangle and the upper part of the auricle are aligned, parallel to the ground and 90 degrees between the head and neck) and with a mechanical stadiometer by providing an upright posture (14). The WHO AnthroPlus program was used to calculate children's height-for-age z scores, BMI, and BMI-for-age z scores (16). The measurement of the waist circumference (cm) of the children was carried out in a standing, upright position, with the feet side by side and the arms free on the sides, in mild expiration, with a tape measure that did not stretch to pass through the midpoint of the distance between the lowest rib bone and the iliac crest and was parallel to the ground (14).

### Evaluation of biochemical findings

The biochemical test results of the children diagnosed with IR were retrieved from the hospital laboratory database. To ensure consistency and reliability, only results obtained within 3 months prior to study enrollment and from the same accredited hospital laboratory were included in the analysis. The following parameters were recorded and evaluated: fasting insulin (μU/mL), fasting blood glucose (mg/dL), HOMA-IR, triglycerides (mg/dL), total cholesterol (mg/dL), Aspartate Aminotransferase (AST, U/L), Alanine Aminotransferase (ALT, U/L), platelet count (103/uL), neutrophil (103/uL), and lymphocyte (103/uL).

The presence of IR in children was determined using the HOMA-IR, calculated as “fasting glucose (mg/dL) × fasting insulin (μU/mL) / 405” (17). A HOMA-IR cut-off value of 3.16, as proposed by Keskin et al. (18), was used to classify participants as IR.

### Systemic Immune Inflammation Index (SII)

The Systemic Immune-Inflammation Index (SII), developed in 2014, was used to assess the immune and inflammatory status of children diagnosed with IR. SII, an inflammatory biomarker, was calculated with the formula “platelet count x neutrophil count/ lymphocyte count” (19).

### Dietary intake record

The researcher collected two-day dietary intake records on weekdays and weekends to assess the total energy and nutrient intake of children. To accurately and reliably evaluate the amount of food consumed by children while taking food consumption records, the book “Food and Food Photography Catalog: Measurements and Quantities” was used (20). The Standard Meal Tariffs book was used to determine the grammages of various ingredients in the meals consumed by children outside the home (21).

The phytochemical and inflammatory indices of the children's diets were calculated from food consumption records, along with the total energy and nutrients they received daily. The Nutrition Information System (BeBiS) 8.2 program was utilized for the calculations (22). The daily energy and nutrient intake requirements of children were also calculated based on the Recommended Dietary Allowances (RDA) specific to their age and gender (23).

### Mediterranean Diet Quality Index (KIDMED)

KIDMED, developed by Serra-Majem et al. (24) and validated in Turkish by Sahingöz et al. (25), was used to assess children's adherence to the Mediterranean diet. A KIDMED total score of ≤ 3 was evaluated as low diet quality, 4–7 as moderate diet quality, and ≥8 as good diet quality.

### Dietary Phytochemical Index (DPI)

An analysis of dietary intake records in the BeBiS program was conducted to determine the total phytochemical intake of children. The “Phytochemical Index (PI)” method developed (26) by McCarty in 2004 was used. With the PI method, the percentage of energy from foods rich in phytochemical compounds in daily energy intake is determined, and this index is calculated with the formula PI= Energy from foods rich in phytochemical content (kcal/day)/Total energy intake (kcal/day) × 100 (26).

### Dietary Inflammatory Index (DII)

An analysis of the dietary intake records in the BeBiS program was conducted to assess the inflammatory potential of children's diets. The “Dietary Inflammatory Index (DII)” calculation method developed by Shivappa et al. (27) in 2014 was applied, and 35 nutrient parameters were evaluated. B-carotene, trans fatty acids, eugenol, flavan-3-ol, flavones, flavonols, flavonones, isoflavones, anthocyanidin, and alcohol not consumed in this age range were not evaluated in this study. The categorization of DII scores into low, medium, or high lacks a universally accepted cut-off. It can be posited that the child's diet is anti-inflammatory if the DII score is low and pro-inflammatory if the score is high (27).

### Pittsburgh Sleep Quality Index (PSQI)

The Pittsburgh Sleep Quality Index (PSQI) was used to assess children's sleep quality. The PSQI was developed by Buysse et al. (28) in 1989, and its validity and reliability were studied in Türkiye by Agargün et al. (29) in 1996. A total PSQI score of < 5 is indicative of “good” sleep quality, while a score of ≥5 is indicative of “bad” sleep quality (28).

### Children's Depression Inventory (CDI)

The Children's Depression Inventory (CDI) was used to evaluate the depression levels of children (30). The validity and reliability study in Turkey was conducted by Öy in 1991 (31). Children with a total CDI score of < 19 had a “low” level of depression, and those with ≥19 had a “high” level of depression (30, 32).

### Statistical evaluation of data

SPSS 27.0 (Statistical Package for Social Sciences) for Windows was used to analyze the research data. The Shapiro-Wilk test was used to assess whether the continuous variables conformed to normality. Since normal distribution was not observed, non-parametric analyses were selected. Descriptive statistics for variables are stated as mean ± standard deviation, median (lower-upper). Descriptive statistics of categorical variables were reported with *n* (%). Independent comparisons between the two groups were made with the Mann-Whitney U test. Pearson chi-square test was used for intergroup comparisons of categorical and 2^*^2 quota tables, and Fisher Freeman Halton test was used for intergroup comparisons of R^*^C quota tables. The Spearman correlation test was performed in the comparison of two continuous variables. *p* < 0.05 was considered statistically significant.

## Results

While 46.7% of the children included in the case group were boys (14 children) and 53.3% were girls (16 children), 33.3% of the children included in the control group were boys (10 children) and 66.7% were girls (20 children). Gender, age groups, and mean ages did not differ significantly between the groups (*p* > 0.05). The statistical similarity (*p* > 0.05) in socioeconomic distributions between the two groups further suggests that the observed differences in nutritional and psychological parameters are more likely related to IR status rather than demographic imbalances. In addition to the diagnosis of IR, it was determined that most of the children (26.7%) who were diagnosed with another disease (23.3%) were diagnosed with fatty liver (Table 1).

**Table 1:** Demographic characteristics, dietary habits, and nutritional supplement use of children.

Variables	Categories	Case group (*n* = 30)	Control group (*n* = 30)	Total (*n* = 60)	*p*	*p*
**Gender**	**Age group**	***n*** **(%)**	***n*** **(%)**	***n*** **(%)**		
Man	10–13 years	9 (64.3)	6 (60.0)	15 (62.5)	1,000^a^	0.292^d^
	14–18 years	5 (35.7)	4 (40.0)	9 (37.5)		
	**Total**	14 (100.0)	10 (100.0)	24 (100.0)		
Woman	10–13 years	6 (37.5)	12 (60.0)	18 (50.0)	0.180^b^	
	14–18 years	10 (62.5)	8 (40.0)	18 (50.0)		
	**Total**	16 (100.0)	20 (100.0)	36 (100.0)		
		**X** ±**SD (Min-Max)**	**X** ±**SD (Min-Max)**	**X** ±**SD (Min-Max)**	***p***	
**Age (years)**		13.63 ± 2.32 13.5(10–17)	13.40 ± 2.04 13(10–17)	13.52 ± 2.17 13(10–17)	0.817^c^	
**Demographic characteristics**		***n*** **(%)**	***n*** **(%)**	***n*** **(%)**	***p***	
Mother's education level						
Illiterate		0 (0.0)	2 (6.7)	2 (3.3)	0,327^a^	
Primary school		11 (36.7)	6 (20.0)	17 (28.3)		
Middle school		5 (16.7)	3 (10.0)	8 (13.3)		
High school		7 (23.3)	11 (36.7)	18 (30.0)		
University		7 (23.3)	8 (26.7)	15 (25.0)		
Father's education level						
Illiterate		0 (0.0)	1 (3.3)	1 (1.7)	0,773^a^	
Primary school		7 (23.3)	5 (16.7)	12 (20.0)		
Middle school		2 (6.7)	4 (13.3)	6 (10.0)		
High school		13 (43.3)	11 (36.7)	24 (40.0)		
University		8 (26.7)	9 (30.0)	17 (28.3)		
Income status of the family						
At minimum wage		4 (13.3)	2 (6.7)	6 (10.0)	0.647^a^	
Above minimum wage		7 (23.3)	11 (36.7)	18 (30.0)		
Twice the minimum wage		8 (26.7)	7 (23.3)	15 (25.0)		
More than twice the minimum wage		11 (36.7)	10 (33.3)	21 (35.0)		
Having been diagnosed with another disease						
Yes		8 (26.7)	-	8 (26.7)	**-**	
No		22 (73.3)	-	22 (73.3)		
Diseases diagnosed ^*^						
Fatty liver		7 (23.3)	**-**	7 (23.3)		
PCOS		1 (3.3)	**-**	1 (3.3)		
GIS Diseases		1 (3.3)	**-**	1 (3.3)		
**Dietary habits and supplement use**		***n*** **(%)**	***n*** **(%)**	***n*** **(%)**	**X** ^2^	***p***
Number of main meals						
2 main meals		9 (30.0)	6 (20.0)	15 (25.0)	0.800	0.371^e^
3 main meals		21 (70.0)	24 (80.0)	45 (75.0)		
Skipped the main meal						
Breakfast		7 (23.3)	4 (13.3)	11 (18.3)	1.002	0.317^a^
Dinner		2 (6.7)	2 (6.7)	4 (6.7)	0.000	1,000^a^
Nutritional supplements usage						
No		19 (63.3)	18 (60.0)	37 (61.7)	0.071	0.791^b^
Yes		11 (36.7)	12 (40.0)	23 (38.3)		
Nutritional supplements^*^						
B-group vitamins		4 (13.3)	7 (23.3)	11 (18.3)	1.002	0.317^a^
Vitamin C		0 (0.0)	2 (6.7)	2 (3.3)	2.069	0.492^a^
Vitamin D		8 (26.7)	5 (16.7)	13 (21.7)	0.884	0.347^b^
Iron		1 (3.3)	4 (13.3)	5 (8.3)	1,964	0.353^a^
Zinc		0 (0.0)	2 (6.7)	2 (3.3)	2.069	0.492^a^
Plant extracts		1 (3.3)	0 (0.0)	1 (1.7)	1.017	1,000^a^
Omega_3		2 (6.7)	1 (3.3)	3 (5.0)	0.351	1,000^a^

^a^ Fisher exact test, ^b^ Pearson chi-square test, ^c^ Mann-Whitney U test, ^d^ Intergroup sex, ^e^ Fisher-Freeman-Halton test, Pearson chi-square test. ^*^More than one option is marked. GIS, Gastrointestinal System; PCOS, Polycystic Ovary Syndrome.

Table 2 shows the anthropometric measurements of children by group and age. It was observed that the children in the case group had significantly higher body weights (91.76 ± 25.76 kg and 53.83 ± 13.03 kg, respectively), BMI (34.72 ± 7.81 kg/m^2^ and 21.05 ± 3.38 kg/m^2^, respectively), waist circumferences (104.77 ± 13.43 cm and 73.23 ± 8.82 cm, respectively), body fat ratios (41.98 ± 7.327 and 23.68 ± 5.363, respectively), and BMI z score by age (3.26 ± 1.05 and 0.53 ± 0.94, respectively) compared to the control group (*p* < 0.05). It was found that children aged 10–13 in the case group had significantly higher body weight, BMI, waist circumference, body fat percentage, and age-appropriate BMI z-score values compared to children aged 10–13 in the control group, and also that children aged 14–18 in the case group had significantly higher values compared to children aged 14–18 in the control group (*p* < 0.05). According to the body fat ratio classification, it was determined that 93.3% of the children in the case group were obese, while 80%of the children in the control group were normal and, there was a significant difference between the groups (*p* < 0.05) (Not shown in the table).

**Table 2:** Anthropometric measurements of children by groups and age groups.

Anthropometric measurements	Case group	Case group	Case group	Case group	Control group	Control group	Control group	Control group			
	**10–13 years**	**14–18 years**	**Total (*****n*** = **30)**		**10–13 years**	**14–18 years**	**Total (*****n*** = **30)**				
	**X** ±**SD Median (Min-Max)**	**X** ±**SD Median (Min-Max)**	**X** ±**SD Median (Min-Max)**	p*^c^	**X** ±**SD Median (Min-Max)**	**X** ±**SD Median (Min-Max)**	**X** ±**SD Median (Min-Max)**	p*^c^	**P** ^c^	**P1** ^c^	**P2** ^c^
Body weight (kg)	78.14 ± 18.84 81.9 (50.2–106.0)	105.38 ± 24.94 105.0 (77.2–175.0)	91.76 ± 25.76 91.5 (50.2–175.0)	**0.003**	48.68 ± 10.78 48.1 (29.5–70.7)	61.56 ± 12.63 58.0 (46.9–84.1)	53.83 ± 13.03 52.2 (29.5–84.1)	**0.018**	**0.001**	**< 0.001**	**< 0.001**
Height (cm)	158.80 ± 7.58 158.0 (145.0–174.0)	164.53 ± 7.20 163.0 (150.0–178.0)	161.67 ± 7.82 163.0 (145.0–178.0)	0.052	153.56 ± 8.06 152.5 (140.0–168.0)	167.08 ± 6.41 166.0 (157.0–177.0)	158.97 ± 9.96 159.0 (140.0–177.0)	**< 0.001**	0.317	0.085	0.434
BMI (kg/m^2^)	30.64 ± 5.49 30.8 (22.3–39.9)	38.79 ± 7.79 37.6 (29.4–59.2)	34.72 ± 7.81 33.4 (22.3–59.2)	**0.004**	20.49 ± 3.41 20.4 (15.1–28.0)	21.89 ± 3.28 21.5 (18.2–29.1)	21.05 ± 3.38 21.1 (15.1–29.1)	0.162	**< 0.001**	**< 0.001**	**< 0.001**
Waist circumference (cm)	98.80 ± 11.88 102.0 (77.0–118.0)	110.73 ± 12.50 110.0 (90.0–137.0)	104.77 ± 13.43 104.5 (77.0–137.0)	**0.020**	72.17 ± 8.24 71.5 (61.0–94.0)	74.83 ± 9.77 71.0 (64.0–95.0)	73.23 ± 8.82 71.0 (61.0–95.0)	0.445	**< 0.001**	**< 0.001**	**< 0.001**
Body fat ratio (%)	39.91 ± 5.31 38.5 (30.9–50.6)	44.06 ± 8.58 41.2 (31.7–62.9)	41.98 ± 7.32 40.9 (30.9–62.9)	0.178	22.84 ± 5.10 22.5 (13.7–30.7)	24.94 ± 5.71 23.5 (18.8–40.0)	23.68 ± 5.36 23.4 (13.7–40.0)	0.485	**< 0.001**	**< 0.001**	**< 0.001**
Height for age Z score	1.23 ± 0.93 1.1 (−0.4–2.7)	−0.16 ± 0.90 −0.2 (−2.0–1.4)	0.54 ± 1.15 0.8 (−2.0–2.7)	**0.001**	0.16 ± 0.89 0.2 (−1.2–2.2)	0.17 ± 0.71 0.2 (−0.8–2.0)	0.16 ± 0.81 0.2 (−1.2–2.2)	0.949	0.126	**0.002**	0.354
BMI Z score by age	3.01 ± 0.90 2.9 (1.4–4.9)	3.50 ± 1.16 3.2 (2.2–6.6)	3.26 ± 1.05 3.1 (1.4–6.6)	0.309	0.67 ± 0.97 0.7 (−1.0–2.6)	0.32 ± 0.90 0.2 (−0.9–2.2)	0.53 ± 0.94 0.4 (−1.0–2.6)	0.330	**< 0.001**	**< 0.001**	**< 0.001**

p^*^, Difference between in-group age groups; P, (total) difference between groups; P1, case-control 10–13 years; P2, difference between case-control 14–18 years; ^c^ Mann-Whitney U test. The values in bold indicate that *p* < 0.05, which is statistically significant.

It was determined that 36.7% of the children in the case group and 63.3% in the control group engaged in physical activity, and there was a significant difference between the groups in physical activity status (*p* = 0.039). However, there was no significant difference between the groups in terms of the frequency of children's physical activity (*p* > 0.05) (Table 3).

**Table 3:** Physical activity status and frequency of children by group.

Physical activity	Categories	Case group (*n* = 30)	Control group (*n* = 30)	Total (*n* = 60)	X^2^	*p*
		***n*** **(%)**	***n*** **(%)**	***n*** **(%)**		
State of engaging in physical activity	Yes	11 (36.7)	19 (63.3)	30 (50.0)	4.267	**0.039** ^**b**^
	No	19 (63.3)	11 (36.7)	30 (50.0)		
Frequency of physical activity	Doesn't	19 (63.3)	11 (36.7)	30 (50.0)	5.734	0.213^d^
	Less than 1 h per week	1 (3.3)	4 (13.3)	5 (8.3)		
	1 h a week	1 (3.3)	2 (6.7)	3 (5.0)		
	2 h a week	6 (20.0)	6 (20.0)	12 (20.0)		
	3 h or more per week	3 (10.0)	7 (23.3)	10 (16.7)		

^b^ Pearson chi-square test, ^d^ Fisher-Freeman-Halton test. The values in bold indicate that *p* < 0.05, which is statistically significant.

Table 4 shows the daily energy and macronutrient intake of children by group and age, along with their intake percentages according to the RDA. The energy (kcal), carbohydrate (g), protein (g), fat (g), saturated fatty acids (g), polyunsaturated fatty acids (g), and cholesterol (mg) intakes of children in the case group, as well as the ratios of energy (kcal), carbohydrate (g), protein (g), and fat (g) intakes to RDA, were found to be significantly higher than those of children in the control group (*p* < 0.05). When examined by age group within each group, there was no statistically significant difference in energy and macronutrient intakes or RDA fulfillment rates between the 10–13 and 14–18 age groups in the case and control groups (*p* > 0.05). Energy (kcal), carbohydrate (g), protein (g), fat (g), saturated fatty acids (g), polyunsaturated fatty acids (g), and cholesterol (mg) intake was found to be statistically significantly higher in children aged 10–13 in the case group compared to children aged 10–13 in the control group (*p* < 0.05). Additionally, children aged 14–18 years in the case group had significantly higher intake of thiamine (mg), niacin (mg), vitamin B6 (mg), iron (mg), magnesium (mg), sodium (mg), and phosphorus (mg) intake were significantly higher in children aged 14–18 years in the case group compared to those in the control group (*p* < 0.05) (Supplementary Table 1).

**Table 4:** Daily energy and macronutrient intake of children by group and age group, and the percentage of RDA met.

Energy and macronutrients	Case group	Case group	Case group	Case group	Control group	Control group	Control group	Control group			
	**10–13 years**	**14–18 years**	**Total (*****n*** = **30)**		**10–13 years**	**14–18 years**	**Total (*****n*** = **30)**				
	**X** ±**SD Median (Min-Max)**	**X** ±**SD Median (Min-Max)**	**X** ±**SD Median (Min-Max)**	p*^c^	**X** ±**SD Median (Min-Max)**	**X** ±**SD Median (Min-Max)**	**X** ±**SD Median (Min-Max)**	p*^c^	**P** ^c^	**P1** ^c^	**P2** ^c^
Energy (kcal)	2846.04 ± 413.69 2929.6 (1759.8–3322.3)	2840.26 ± 545.05 2664.3 (2006.0–3713.3)	2843.15 ± 475.44 2833.6 (1759.8–3713.3)	0.820	1916.67 ± 421.62 1959.5 (1140.2–2735.0)	1860.32 ± 474.42 1975.5 (1079.4–2762.6)	1894.13 ± 436.31 1968.7 (1079.4–2762.6)	0.899	**< 0.001**	**< 0.001**	**< 0.001**
Energy (%RDA)	147.27 ± 21.37 151.0 (91.0–172.0)	146.80 ± 28.15 138.0 (104.0–192.0)	147.03 ± 24.55 146.5 (91.0–192.0)	0.771	99.17 ± 21.71 101.5 (59.0–141.0)	96.17 ± 24.42 102.0 (56.0–143.0)	97.97 ± 22.46 102.0 (56.0–143.0)	0.849	**< 0.001**	**< 0.001**	**< 0.001**
Carbohydrate (g)	289.69 ± 58.87 301.6 (176.4–426.0)	298.71 ± 75.37 262.7 (167.9–396.6)	294.20 ± 66.61 296.7 (167.9–426.0)	0.803	192.63 ± 58.16 186.0 (96.8–294.7)	173.88 ± 60.47 178.9 (71.9–285.9)	185.13 ± 58.80 182.6 (71.9–294.7)	0.525	**< 0.001**	**< 0.001**	**< 0.001**
Carbohydrate (%)	41.53 ± 5.26 39.0 (34.0–52.0)	42.67 ± 4.13 43.0 (34.0–48.0)	42.10 ± 4.68 42.0 (34.0–52.0)	0.288	41.06 ± 8.04 41.0 (28.0–53.0)	37.42 ± 6.30 38.5 (24.0–45.0)	39.60 ± 7.50 39.5 (24.0–53.0)	0.227	0.151	0.928	**0.019**
Carbohydrate (%RDA)	104.87 ± 21.34 109.0 (64.0–154.0)	108.27 ± 27.38 95.0 (61.0–144.0)	106.57 ± 24.18 107.5 (61.0–154.0)	0.787	69.78 ± 21.03 67.0 (35.0–107.0)	63.00 ± 21.95 64.5 (26.0–104.0)	67.07 ± 21.29 66.0 (26.0–107.0)	0.553	**< 0.001**	**< 0.001**	**< 0.001**
Protein (g)	94.49 ± 13.10 94.3 (76.9–116.5)	93.85 ± 20.14 92.2 (56.7–129.5)	94.17 ± 16.69 93.7 (56.7–129.5)	0.852	66.42 ± 20.47 64.4 (38.1–112.3)	69.67 ± 20.59 63.4 (45.5–110.9)	67.72 ± 20.22 64.4 (38.1–112.3)	0.767	**< 0.001**	**< 0.001**	**0.006**
Protein (%)	13.80 ± 2.21 14.0 (10.0–19.0)	13.47 ± 1.45 13.0 (10.0–16.0)	13.63 ± 1.84 14.0 (10.0–19.0)	0.497	14.11 ± 2.86 13.5 (10.0–20.0)	15.25 ± 1.71 15.5 (12.0–18.0)	14.57 ± 2.50 14.5 (10.0–20.0)	0.200	0.108	0.798	**0.009**
Protein (%RDA)	165.47 ± 22.82 165.0 (135.0–204.0)	164.20 ± 35.31 161.0 (99.0–227.0)	164.83 ± 29.22 164.0 (99.0–227.0)	0.868	116.33 ± 35.79 112.5 (67.0–197.0)	122.00 ± 36.01 111.0 (80.0–194.0)	118.60 ± 35.37 112.5 (67.0–197.0)	0.783	**< 0.001**	**< 0.001**	**0.006**
	**X** ±**SD Median (Min-Max)**	**X** ±**SD Median (Min-Max)**	**X** ±**SD Median (Min-Max)**	p*^c^	**X** ±**SD Median (Min-Max)**	**X** ±**SD Median (Min-Max)**	**X** ±**SD Median (Min-Max)**	p*^c^	**P** ^c^	**P1** ^c^	**P2** ^c^
Fat (g)	143.40 ± 28.86 149.8 (79.4–185.0)	139.07 ± 23.15 137.7 (101.7–182.6)	141.23 ± 25.80 144.1 (79.4–185.0)	0.443	95.72 ± 23.20 97.1 (56.3–128.0)	96.88 ± 19.85 97.8 (66.9–128.3)	96.19 ± 21.57 97.2 (56.3–128.3)	0.933	**< 0.001**	**< 0.001**	**< 0.001**
Fat (%)	44.73 ± 5.45 47.0 (32.0–52.0)	43.87 ± 4.24 44.0 (36.0–51.0)	44.30 ± 4.82 44.5 (32.0–52.0)	0.466	44.61 ± 6.55 44.0 (35.0–56.0)	47.17 ± 5.71 45.0 (41.0–60.0)	45.63 ± 6.26 44.0 (35.0–60.0)	0.328	0.563	0.885	0.191
Fat (%RDA)	218.53 ± 43.95 228.0 (121.0–282.0)	211.93 ± 35.23 210.0 (155.0–278.0)	215.23 ± 39.28 219.5 (121.0–282.0)	0.443	145.83 ± 35.30 148.0 (86.0–195.0)	147.67 ± 30.22 149.0 (102.0–196.0)	146.57 ± 32.83 148.0 (86.0–196.0)	0.899	**< 0.001**	**< 0.001**	**< 0.001**
Saturated fatty acids (g)	46.46 ± 12.31 48.5 (24.9–75.3)	42.93 ± 11.15 42.8 (27.1–62.7)	44.69 ± 11.68 45.3 (24.9–75.3)	0.310	30.38 ± 9.66 31.7 (14.8–49.6)	33.20 ± 8.93 34.4 (15.4–43.8)	31.51 ± 9.33 31.8 (14.8–49.6)	0.280	**< 0.001**	**0.001**	**0.019**
Monounsaturated fatty acids (g)	42.09 ± 11.03 42.0 (21.1–59.9)	38.05 ± 9.49 38.0 (18.7–53.1)	40.07 ± 10.32 41.6 (18.7–59.9)	0.281	37.66 ± 14.01 39.2 (16.3–69.1)	33.14 ± 9.97 32.5 (18.4–52.5)	35.85 ± 12.56 36.3 (16.3–69.1)	0.397	0.114	0.270	0.164
Polyunsaturated fatty acids (g)	23.69 ± 9.22 23.2 (11.8–41.8)	25.36 ± 8.42 27.9 (5.3–36.2)	24.52 ± 8.72 26.2 (5.3–41.8)	0.407	12.69 ± 5.72 12.1 (4.4–24.7)	14.63 ± 7.07 15.3 (4.7–26.4)	13.47 ± 6.25 12.5 (4.4–26.4)	0.069	**< 0.001**	**0.001**	**0.003**
Cholesterol (mg)	501.82 ± 134.05 524.6 (283.1–712.5)	459.05 ± 172.47 486.7 (125.6–765.6)	480.44 ± 153.33 501.4 (125.6–765.6)	0.351	275.61 ± 130.05 243.3 (89.7–487.2)	387.31 ± 169.94 375.7 (81.0–709.2)	320.29 ± 154.81 311.8 (81.0–709.2)	0.352	**< 0.001**	**< 0.001**	0.306

p^*^, Difference between in-group age groups; P, (total) difference between groups; P1, difference between case-control 10–13 years; P2, difference between case-control 14–18 years; ^c^ Mann-Whitney U test. The values in bold indicate that *p* < 0.05, which is statistically significant.

Table 5 shows the KIDMED, DPI, DII, PSQI, and CDI scores for the children across and age. Compared to the children in the case group, the KIDMED (2.40 ± 1.61 and 6.17 ± 2.29, respectively) and DPI (9.67 ± 5.52 and 24.01 ± 12.27, respectively) scores of the children in the control group were found to be statistically significantly higher (*p* < 0.05). There was no statistically significant difference between the groups in DII score (*p* > 0.05). The total PSQI and CDI scores of the children in the case group (6.60 ± 2.56 and 14.43 ± 6.30, respectively) were found to be statistically significantly higher than those in the control group (3.67 ± 1.86 and 10.17 ± 5.40, respectively) (*p* < 0.05). The mean SII of the children in the case group was 646.53 ± 419.17; that of the children between the ages of 10–13 was 677.37 ± 529.94, and that of the children between the ages of 14–18 was 615.68 ± 284.74 (not shown in the table).

**Table 5:** KIDMED, DPI, DII, PSQI, and CDI scores of children by groups and age groups.

Points	Case group	Case group	Case group	Case group	Control group	Control group	Control group	Control group			
	**10–13 years**	**14–18 years**	**Total (*****n*** = **30)**		**10–13 years**	**14–18 years**	**Total (*****n*** = **30)**				
	**X** ±**SD Median (Min-Max)**	**X** ±**SD Median (Min-Max)**	**X** ±**SD Median (Min-Max)**	p*^c^	**X** ±**SD Median (Min-Max)**	**X** ±**SD Median (Min-Max)**	**X** ±**SD Median (Min-Max)**	p*^c^	**P** ^c^	**P1** ^c^	**P2** ^c^
KIDMED	2.87 ± 1.35 3.0 (0.0–5.0)	1.93 ± 1.75 2.0 (−2.0–4.0)	2.40 ± 1.61 3.0 (−2.0–5.0)	0.163	6.67 ± 2.22 7.0 (1.0–10.0)	5.42 ± 2.27 5.5 (2.0–9.0)	6.17 ± 2.29 6.0 (1.0–10.0)	0.129	**< 0.001**	**< 0.001**	**0.001**
DPI	10.18 ± 4.53 9.0 (2.2–17.2)	9.16 ± 6.49 7.2 (2.1–27.6)	9.67 ± 5.52 8.5 (2.1–27.6)	0.290	27.24 ± 10.91 27.3 (1.3–45.4)	19.16 ± 13.04 13.7 (7.1–44.5)	24.01 ± 12.27 22.5 (1.3–45.4)	**0.038**	**< 0.001**	**< 0.001**	**0.011**
DII	2.25 ± 1.15 2.1 (0.3–4.2)	1.93 ± 1.51 2.1 (−0.3–4.2)	2,09 ± 1,33 2.1 (−0.3–4.2)	0.575	2.22 ± 1.82 2.2 (−1.7–5.0)	2.57 ± 1.58 2.6 (−0.7–5.4)	2.36 ± 1.71 2.4 (−1.7–5.4)	0.553	0.487	1,000	0.354
PSQI	5.60 ± 1.80 5 (3–10)	7.60 ± 2.87 7 (3–11)	6.60 ± 2.56 6 (3–11)	**0.033**	3.00 ± 1.08 3 (1–5)	4.67 ± 2.34 4 (2–10)	3.67 ± 1.86 4 (1–10)	**0.026**	**< 0.001**	**< 0.001**	**0.010**
CDI	11.87 ± 3.85 12.0 (4.0–18.0)	17.00 ± 7.30 16.0 (8.0–32.0)	14.43 ± 6.30 13.0 (4.0–32.0)	**0.041**	9.61 ± 4.69 10.0 (2.0–18.0)	11.00 ± 6.46 9.0 (4.0–26.0)	10.17 ± 5.40 9.5 (2.0–26.0)	0.915	**0.008**	0.186	**0.025**

p^*^, Difference between in-group age groups; P, (Total) difference between groups; P1, 10–13 years old, difference between case-control; P2, 14–18 years old, difference between case-control; ^c^ Mann-Whitney U test; CDI, Children's Depression Inventory; DII, Dietary Inflammatory Index; DPI, Dietary Phytochemical Index; KIDMED, Mediterranean Diet Quality Index; PSQI, Pittsburgh Sleep Quality Index. The values in bold indicate that *p* < 0.05, which is statistically significant.

When examined according to age groups within the groups, it was determined that the total PSQI and CDI scores of the children between the ages of 14–18 in the case group were higher than the scores of the children between the ages of 10–13, showing a statistically significant difference (*p* < 0.05). In the control group, the total PSQI scores of the children aged 14–18 were significantly higher than those of the children aged 10–13 (*p* < 0.05).

The biochemical findings in the case group, along with their correlation with specific parameters, are presented in Table 6. A significant positive correlation was found between children's fasting insulin levels and HOMA-IR levels (r = 0.934, *p* < 0.05). A significant positive correlation was found between energy (kcal) intake and fasting insulin and HOMA-IR levels (r = 0.461, *p* = 0.010; r = 0.448, *p* = 0.013, respectively). A significant positive correlation was found between carbohydrate (g) intake and fasting insulin and HOMA-IR values (r = 0.410, *p* = 0.024; r = 0.380, *p* = 0.039, respectively); and between protein (g) intake and fasting insulin and HOMA-IR values (r = 0.444, *p* = 0.014; r = 0.423, *p* = 0.020, respectively). A significant positive correlation was found between fat (g) intake and HOMA-IR values (r = 0.375, *p* = 0.041).

**Table 6:** Correlation of biochemical findings of children in the case group with various parameters.

Biochemical findings	*r*/*p*	Fasting insulin (μU/mL)	Fasting blood glucose (mg/dl)	HOMA-IR	Energy (kcal)	Carbohydrate (g)	Protein (g)	Fat (g)	KIDMED	DPI	DII	PSQI	CDI
Fasting insulin	r	1	−0.203	0.934	0.461	0.410	0.444	0.359	−0,367	−0.626	0.011	0.284	0.593
	*p*	.	0.282	**< 0.001**	**0.010**	**0.024**	**0.014**	0.051	**0.046**	**< 0.001**	0.953	0.128	**0.001**
Fasting blood glucose	r	−0.203	1	0.088	−0.032	−0.026	0.033	−0,001	0.188	0.264	−0.139	−0.096	−0.003
	*p*	0.282	.	0.645	0.868	0.891	0.862	0.995	0.320	0.159	0.462	0.614	0.989
HOMA-IR	r	0.934	0.088	1	0.448	0.380	0.423	0.375	−0,267	−0.516	−0.080	0.202	0.556
	*p*	**< 0.001**	0.645	.	**0.013**	**0.039**	**0.020**	**0.041**	0.153	**0.003**	0.674	0.285	**0.001**
Triglyceride (mg/dl)	r	0.059	−0.252	0.017	0.103	0.169	−0.082	0.060	−0.234	−0,224	−0.028	0.258	0.041
	*p*	0.758	0.179	0.927	0.589	0.371	0.667	0.753	0.213	0.233	0.884	0.168	0.830
Total cholesterol (mg/dL)	r	−0.034	0.045	0.053	−0.135	−0.196	−0,354	−0.170	−0.038	−0.097	0.149	0.009	−0.072
	*p*	0.860	0.814	0.781	0.478	0.300	0.055	0.370	0.841	0.608	0.432	0.961	0.705
AST (U/L)	r	−0.075	−0.414	−0.213	−0.128	−0.062	−0,150	−0.187	−0.099	−0.143	−0.020	0.182	−0.238
	*p*	0.693	**0.023**	0.259	0.500	0.744	0.427	0.321	0.604	0.452	0.915	0.336	0.205
ALT (U/L)	r	0.121	−0.402	0.000	0.096	0.095	0.118	0.031	−0.237	−0.213	−0,150	0.286	−0.147
	*p*	0.524	**0.028**	1,000	0.612	0.617	0.535	0.873	0.207	0.258	0.428	0.125	0.440
Platelets (10^3^/uL)	r	−0.008	0.177	0.012	−0.022	0.013	0.004	−0.114	−0,100	−0.237	−0.040	0.192	0.111
	*p*	0.965	0.349	0.949	0.906	0.946	0.984	0.547	0.601	0.207	0.834	0.308	0.560
Neutrophil (10^3^/uL)	r	0.149	0.051	0.135	−0.053	−0.147	−0.161	0.166	−0.202	−0,227	0.146	0.103	0.342
	*p*	0.432	0.790	0.477	0.781	0.437	0.395	0.381	0.284	0.227	0.441	0.589	0.065
Lymphocyte (10^3^/uL)	r	−0.171	−0.155	−0.306	−0.136	−0.079	−0.085	−0.252	−0.198	−0.257	0.108	0.404	0.060
	*p*	0.366	0.413	0,100	0.473	0.677	0.656	0.179	0.294	0.171	0.571	**0.027**	0.752
SII	r	0.142	0.227	0.211	0.019	−0.045	−0.043	0.182	−0.114	−0.152	0.032	−0.021	0.262
	*p*	0.455	0.227	0.264	0.921	0.812	0.822	0.335	0.548	0.424	0.867	0.914	0.162

Spearman correlation test; ALT (U/L), Alanine Aminotransferas; AST (U/L), Aspartate Aminotransferase; CDI, Children's Depression Inventory; DII, Dietary Inflammatory Index; DPI, Dietary Phytochemical Index; HOMA-IR, Homeostatic Model Assessment of Insulin Resistance; KIDMED, Mediterranean Diet Quality Index; PSQI, Pittsburgh Sleep Quality Index; SII, Systemic Immune-Inflammation Index. The values in bold indicate that *p* < 0.05, which is statistically significant.

A significant negative correlation was found between children's fasting insulin levels and their KIDMED and DFI scores (r = −0.367, *p* = 0.046; r = −0.626, *p* < 0.05, respectively). A significant positive correlation was found between fasting insulin levels and the CDO score (r = 0.593, *p* = 0.001). A significant negative correlation was found between HOMA-IR values and DFI scores (r = −0.516, *p* = 0.003). A significant positive correlation was found between HOMA-IR values and CDO scores (r = 0.556, *p* = 0.001). A significant positive correlation was found between lymphocyte values and PUKI scores (r = 0.404, *p* = 0.027).

## Discussion

This study was conducted to compare the nutritional status, sleep quality, and depression levels of children between the ages of 10 and 18 who were diagnosed with IR with their healthy peers. In addition, the biochemical findings of children diagnosed with IR were evaluated, and the relationships between these findings and the children's SII scores, energy and macronutrient intakes, KIDMED, DPI, DII, PSQI, and CDI scores were examined.

Firstly, in our study, children with IR had significantly higher body weight, BMI, waist circumference, body fat percentage, and BMI z-score by age than healthy children (*p* < 0.05). In some studies conducted with children and adolescents, it was reported that the body weight, BMI, waist circumference, and body fat percentages of children with IR were significantly higher (33, 34). Therefore, our findings, in line with the literature, reveal that IR is closely related to body composition. In addition, obesity is reported to be a leading risk factor for IR, and its prevalence increases with age (35, 36). In the United States, it has been reported that the prevalence of obesity from 2017 to March 2020 was 12.7% in children between the ages of 2–5, 20.7% in children between the ages of 6–11, and 22.2% in adolescents between the ages of 12–19 (36, 37). In our study, children aged 14–18 with IR had significantly higher body weight, BMI, and waist circumference than children aged 10–13 (*p* < 0.05). It is thought that this situation can be explained by the fact that obesity in childhood tends to persist into adulthood. Therefore, it can be said that the increasing prevalence of obesity with advancing age plays a critical role in the prominence of IR, especially in adolescence.

In both groups of children in our study, the percentage of energy from carbohydrates was below the recommended level, while that from fats was above it. It is recommended that 45–60% of the daily energy intake should come from carbohydrates, 20–35% from fats, and 10–20% from proteins (38). Therefore, even if a child is currently healthy, it is important to keep the percentages of energy from carbohydrates, proteins, and fats under control. Otherwise, children who are currently healthy may face obesity and obesity-related diseases later in life.

According to the RDA criteria, nutrient intake was classified as insufficient when ≤ 67%, adequate when 67–133%, and excessive when ≥133% (14). According to this evaluation, both children with IR and healthy children were found to have high fat (g) intake, while their intake of polyunsaturated fatty acids (g) was insufficient in both groups. Therefore, care should be taken to ensure that children with IR and children with good health receive adequate and balanced energy and nutrients, including total fat and healthy fatty acids. The findings of our study emphasize the importance of educating children and their families about adequate and balanced nutrition for children with IR. Additionally, adequate and balanced energy and nutrient intake is necessary to protect healthy children from obesity, IR, hypertension, and cardiovascular disease.

In our study, we found that children with IR had statistically significantly lower mean KIDMED scores than children without IR (*p* < 0.05). Low adherence to the Mediterranean diet has been reported to be associated with an increased risk of developing IR (39). A study of school children in Greece found that poor adherence to the Mediterranean diet increased the likelihood of central obesity, hypertriglyceridemia, and IR (40). Similar to our study, the study by Tunçer et al. (39) on children and adolescents found that the mean KIDMED scores of children and adolescents with IR (4.3 ± 2.72) were significantly lower than those without IR (5.4 ± 2.57). Furthermore, in our study, when we examined KIDMED scores by age group, we found that children in the 10–13 age range in both the case and control groups tended to have higher KIDMED scores, indicating greater adherence to the Mediterranean diet. However, no statistically significant difference was found between the age groups (*p* > 0.05). In a study by Rosi et al. (41), the rate of children with high adherence to the Mediterranean diet was 22.7% among those aged 12–14, and 21.4% among those aged 15–17.

It has been reported that higher consumption of phytochemical-rich foods may protect against the development of IR (42). In a study by Tirani et al. (43), adolescents with higher phytochemical intake were less likely to have metabolically unhealthy overweight/obesity. In our study, it was found that the DPI scores of children with IR were statistically significantly lower than their healthy peers (*p* < 0.05). Therefore, in our study, consistent with the literature, we observed a close relationship between the poverty of children's diets in phytochemicals and the development of IR.

When we examine the inflammatory potential of the diet, a study by Shu et al. (44) found that a pro-inflammatory diet (i.e., a higher DII score) increases the likelihood of developing IR and prediabetes. It has also been reported that promoting an anti-inflammatory diet protects glucose-insulin homeostasis and reduces the likelihood. In a case-control study conducted on adolescent boys in Iran, the relationship between adolescent DII scores and overweight and obesity was examined, and a positive relationship was found between DII and obesity. It was determined that adolescents with a DII score of >0.02 were 1.5 times more likely to be overweight and obese compared to adolescents with a DII score of ≤ 0.02 (45). However, in a study conducted by Rakhman and Oktafiani (46), which aimed to examine the differences between the DII scores of obese and non-obese individuals, it was found that the mean DII score was 2.29 ± 0.74 in the group with normal body weight and 1.32 ± 1.00 in the obese group, and a significant difference was found between the groups in terms of DII score. Karimbeiki et al. (47) found that individuals with normal body weight had higher DII scores than those with obesity. They concluded that normal body weight does not necessarily indicate that a diet has lower inflammatory potential. In our study, it was observed that the DII scores of healthy children tended to be higher than those of children with IR (2.36 ± 1.71 and 2.09 ± 1.33, respectively), but no statistically significant difference was found (*p* < 0.05). It is considered that the control group's tendency to have a more pro-inflammatory dietary intake, reflected by a higher DII score, may be explained by the fact that children with IR generally consume higher amounts of nutrients, including those with anti-inflammatory properties, according to their dietary records. Furthermore, the fact that no significant difference was found between the groups in terms of DII scores can be explained by various factors. The fact that dietary data were self-reported (risk of recall bias and underreporting) and that both groups had similar cultural dietary habits may have prevented a significant difference from emerging between the groups in terms of DII scores. In addition to these factors, the lack of standard cutoff points specific to the pediatric age group for the DII score also makes interpreting the obtained scores difficult. In the literature, contradictory findings regarding DII have been reported, and to clarify this issue, studies with larger sample sizes and longer follow-up periods are required.

In our study, it was found that the median PSQI total scores of children with IR were statistically significantly higher than those without IR (*p* < 0.05). It has been reported that sleep disorders contribute to changes in glucose metabolism and an increase in cardiometabolic risk, and low sleep quality in young people is associated with components of metabolic syndrome, such as dyslipidemia and IR (48). In a case-control study conducted on obese and non-obese adolescents, it was found that there was a significant relationship between sleep quality and obesity. The median PSQI score was found to be significantly higher in the obese group compared to the non-obese group [6(2–16) and 5(2–12), respectively] (49). In addition, in a study by Simşek and Tekgül (50) on adolescents, late bedtime (≥24.00) was more common among late adolescence than those in the earlier age group. In our study, it was found that the total PSQI scores of the children in the 14–18 age group were statistically significantly higher than the scores of the children in the 10–13 age group, both in the case and control group (*p* < 0.05). The literature suggests that this situation is due to pre-sleep tea or coffee consumption, older age groups' later bedtimes during the university exam preparation period, and greater social media use before sleep.

Depression is reported to be a mood disorder that can increase the risk of developing IR and type 2 diabetes, and vice versa (51). In a study of overweight and obese children, it was concluded that IR was more determinant for psychiatric diseases than metabolic comorbidities associated with obesity in young obese patients (52). In our study, similar to the literature, it was found that the CDI scores of children with IR were statistically significantly higher than those of healthy peers (*p* < 0.05). Depression is estimated to occur in 1.4% of adolescents aged 10–14 and 3.5% of those aged 15–19, as reported in the literature (53). In our study, CDI scores were significantly higher in children aged 14–18 compared to those aged 10–13 in the case group (*p* < 0.05). In the control group, scores also tended to be higher in older children, although the difference was not significant (*p* > 0.05). These findings suggest that the increase in depression scores with age may become more pronounced in the presence of conditions such as obesity and IR, possibly due to heightened concerns about physical appearance in older adolescents.

When the nutritional, sleep, and depression findings of our study are considered as a whole, these findings indicate that IR in children should be evaluated not only as a metabolic condition but also in interaction with nutritional habits, sleep quality, and depression status, demonstrating that it is a multidimensional process. Therefore, it can be stated that a more appropriate approach in the assessment of children with IR may be to consider nutrition, sleep, and depression comprehensively, rather than focusing solely on biochemical parameters.

Finally, in the correlation analyses of various parameters, Kostovski et al. (54) reported a positive relationship between HOMA-IR and fasting glucose, insulin, and triglyceride levels in children with IR. Similarly, in our study, a significant relationship was found between HOMA-IR and fasting insulin, which supported the strong link between biochemical indicators of IR. It should be noted that the biochemical correlations observed in this study were calculated exclusively for the IR group. Due to the lack of biochemical data for the control group, these relationships could not be compared with healthy peers, and thus, these findings should be interpreted specifically within the context of pediatric IR. In the literature, it is reported that high energy intake increases IR (55), and the effects of carbohydrate consumption are controversial (56). The fact that energy and carbohydrate intakes were positively associated with HOMA-IR and fasting insulin in our study suggests that this may be due to the high proportion of low-fiber, high-glycemic-index, high-load foods in children's diets. Similarly, protein and fat consumption have been shown to increase IR (57, 58); our results are consistent with these studies in demonstrating a positive relationship between protein and fat intake, and HOMA-IR. In addition, the negative correlation between KIDMED and DPI scores and insulin parameters suggests that a Mediterranean-type diet and a diet rich in phytochemicals may be protective against IR. On the other hand, Hannon et al. (59) reported that insulin parameters increased in the group with high depression scores in obese adolescents; the positive relationship between CDI scores and insulin parameters in our study supports the importance of this psychosocial factor. Although relationships with inflammation markers have been defined in the literature on sleep quality (60), in our study, only a positive relationship was observed between lymphocyte values and PSQI, and no connection was found with insulin parameters. This shows that the sleep-inflammation interaction is more complex, and further studies are needed to clarify it in the context of IR in children. Finally, a pro-inflammatory diet has been reported to be associated with metabolic indicators in Brazilian adolescents (61); however, no significant relationship was observed between DII scores and biochemical parameters in our study. This difference can be explained by cultural and nutritional differences between the populations studied. While our findings reveal significant correlations between HOMA-IR levels and lifestyle factors, these results represent observed associations. Given the non-parametric nature of the statistical tests and the study's design, no definitive conclusions regarding causality can be drawn from these data.

This study has some limitations. Although the case and control groups showed no significant differences in socioeconomic data, recruiting the case group from a clinical setting may imply a higher level of “health-seeking behavior” or “disease awareness” among these families. This awareness could influence the accuracy of self-reported dietary habits and perceptions of psychological wellbeing compared with the school-based control group. Future studies should likely select case-control groups from the same source and perform more detailed matching on these variables. A major limitation of our study is that biochemical parameters were assessed only in the IR group. Since the healthy control group was recruited from schools rather than healthcare facilities, their biochemical data were not available. This prevents a direct comparison of metabolic markers between the two groups and restricts the interpretation of biochemical correlations to the case group alone. Consequently, our findings regarding biochemical markers should be presented descriptively rather than comparatively for children with IR. The fact that the children's food consumption records were collected retrospectively and that the study was conducted on a small sample are among the limitations of the study. Additionally, our study did not assess pubertal status or screen time. It should be considered that these factors may also affect IR, sleep quality, and depression scores. Therefore, these factors should also be taken into consideration in future studies. Finally, due to the study's cross-sectional design and the use of non-parametric comparisons and correlation analyses, causality cannot be inferred from the relationships among the variables. Despite its limitations, this study possesses several significant strengths that contribute to the existing body of pediatric research. First and foremost, it employs a highly comprehensive and multidimensional approach, integrating biological markers (SII, biochemical parameters) with validated indices across nutritional (KIDMED, DPI, DII), sleep (PSQI), and psychological (CDI) domains. The use of internationally recognized and locally validated instruments ensures the reliability and cross-cultural comparability of our findings. Furthermore, the inclusion of a power analysis via G^*^Power for sample size determination strengthens the statistical foundation of the study, ensuring that the detected differences between the case and control groups are not merely due to chance. Lastly, this study is among the few to provide an integrative perspective on how dietary quality and phytochemical intake are simultaneously linked to sleep hygiene and mood disturbances in children with IR, offering a holistic roadmap for future clinical interventions.

## Conclusion

IR is becoming increasingly common in children and adolescents with increasing childhood obesity. Therefore, preventing obesity is the main step in reducing IR. In addition to nutrition and physical activity, sleep and depression factors should also be taken into account. While adequate and quality sleep plays a critical role in the prevention of obesity-related diseases, the more severe course of depression in obese children and adolescents reveals the importance of psychological support.

## Data Availability

The raw data supporting the conclusions of this article will be made available by the authors, without undue reservation.

## Glossary

ALT, Alanine Aminotransferase; AST, Aspartate Aminotransferase; BeBiS, The Nutrition Information System; BMI, Body Mass Index; CDI, Children's Depression Inventory; DII, Dietary Inflammatory Index; DPI, Dietary Phytochemical Index; HOMA-IR, Homeostasis Model Assessment of Insulin Resistance; IR, Insulin Resistance; KIDMED, Mediterranean Diet Quality Index; PI, Phytochemical Index; PSQI, Pittsburgh Sleep Quality Index; RDA, Recommended Dietary Allowances; SII, Systemic Immune-Inflammation Index; SPSS, Statistical Package for Social Sciences; WHO, World Health Organization.
