# Supplementary material for: Beyond metabolism: nutrition, sleep, and psychological wellbeing in children with insulin resistance—a case-control study
Source: Front Nutr. 2026 Mar 30;13:1781309. doi: 10.3389/fnut.2026.1781309 (PMC13071045; doi:10.3389/fnut.2026.1781309)
Supplement: Supplementary file 1 [file Table_1.docx]

**Supplementary Table.** The daily intake of micronutrients among children according to study groups and age groups

| **Micronutrients** | **Case Group (n=30)** | | |  | **Control Group (n=30)** | | |  |  |  |  |
| --- | --- | --- | --- | --- | --- | --- | --- | --- | --- | --- | --- |
|  | **10-13 years** | **14-18 years** | **Total** |  | **10-13 years** | **14-18 years** | **Total** |  |  |  |  |
|  | **X ̄ ± SD**  **Median**  **(Max-Min)** | **X ̄ ± SD**  **Median**  **(Max-Min)** | **X ̄ ± SD**  **Median**  **(Max-Min)** | **p*^c^** | **X ̄ ± SD**  **Median**  **(Max-Min)** | **X ̄ ± SD**  **Median**  **(Max-Min)** | **X ̄ ± SD**  **Median**  **(Max-Min)** | **p*^c^** | **P^c^** | **P1^c^** | **P2^c^** |
| Vitamin A (μg) | 917.69±301.69  826.8  (541.7-1406.4) | 914.39±370.28  896.6  (497.9-1994.7) | 916.04±331.86  861.7  (497.9-1994.7) | 0.663 | 941.22±365.23  946.2  (202.1-1603.4) | 848.14±238.18  845.8  (422.0-1300.8) | 903.99±319.17  885.9  (202.1-1603.4) | 0.498 | 0.859 ^c^ | 0.800 | 0.845 |
| Vitamin D (μg) | 5.25±3.86  4.3  (1.9-17.7) | 4.81±3.48  4.0  (0.7-14.1) | 5.03±3.62  4.2  (0.7-17.7) | 0.648 | 3.81±1.66  3.9  (0.5-6.7) | 4.34±3.25  3.5  (1,4-11,4) | 4.02±2.39  3.5  (0.5-11.4) | 0.703 | 0.294 | 0.328 | 0.591 |
| Vitamin K (μg) | 59.05±36.02  41.6  (18.2-157.3) | 81.19±149.17  36.5  (18.4-611.5) | 70.12±107.22  40.2  (18.2-611.5) | 0.330 | 75.94±82.80  53.8  (10.2-379.0) | 40.18±19.63  37.0  (20.2-77.0) | 61.64±66.95  42.5  (10.2-379.0) | 0.094 | 0.877 | 0.691 | 0.922 |
| Vitamin E (mg) | 20.46±6.87  20.2  (11.1-33.1) | 21.41±8.88  22.1  (5.1-38.2) | 20.94±7.82  21.0  (5.1-38.2) | 0.678 | 12.48±5.08  11.3  (4.2-23.6) | 15.36±8.80  13.1  (5.0-33.4) | 13.63±6.82  12.9  (4.2-33.4) | 0.525 | **<0.001** | **0.001** | 0.075 |
| Vitamin C (mg) | 67.43±36.66  69.2  (25.7-168.2) | 78.26±47.13  59.1  (29.3-177.7) | 72.84±41.85  67.2  (25.7-177.7) | 0.694 | 96.53±50.06  85.6  (22.3-191.2) | 87.43±38.79  89.0  (28.1-166.4) | 92.89±45.39  85.9  (22.3-191.2) | 0.703 | 0.058 | 0.093 | 0.407 |
| Thiamine (mg) | 0.97±0.21  1.0  (0.5-1.3) | 0.95±0.19  1.0  (0.7-1.3) | 0.96±0.20  1.0  (0.5-1.3) | 0.801 | 0.86±0.36  0.8  (0.4-1.6) | 0.73±0.22  0.8  (0.4-1.1) | 0.81±0.31  0.8  (0.4-1.6) | 0.392 | **0.014** | 0.196 | **0.020** |
| Riboflavin (mg) | 1.63±0.36  1.7  (1.0-2.1) | 1.49±0.39  1.5  (0.7-2.3) | 1.56±0.37  1.6  (0.7-2.3) | 0.196 | 1.29±0.53  1.1  (0.6-2.5) | 1.32±0.47  1.1  (0.7-2.1) | 1.30±0.50  1.1  (0.6-2.5) | 0.566 | **0.022** | **0.026** | 0.270 |
| Niacin (mg) | 16.38±3.29  15.2  (13.0-22.5) | 16.84±5.87  16.1  (7.4-27.8) | 16.61±4.68  15.5  (7.4-27.8) | 0.934 | 11.93±5.36  10.7  (5.5-21.9) | 11.19±3.73  11.2  (5.3-16.9) | 11.63±4.71  10.9  (5.3-21.9) | 0.767 | **<0.001** | **0.036** | **0.008** |
| Vitamin B6 (mg) | 1.43±0.33  1.3  (1.1-2.3) | 1.51±0.42  1.4  (0.9-2.5) | 1.47±0.37  1.4  (0.9-2.5) | 0.559 | 1.28±0.49  1.2  (0.5-2.2) | 1.15±0.35  1.1  (0.6-1.9) | 1.23±0.44  1.2  (0.5-2.2) | 0.394 | **0.013** | 0.267 | **0.019** |
| Biotin (μg) | 47.71±12.56  50.6  (27.2-66.6) | 47.63±15.93  47.2  (17.0-81.9) | 47.67±14.09  47.5  (17.0-81.9) | 0.820 | 44.91±18.95  38.7  (19.0-78.3) | 44.05±15.32  41.4  (20.8-70.0) | 44.57±17.31  39.3  (19.0-78.3) | 0.949 | 0.284 | 0.539 | 0.305 |

**Supplementary Table.** The daily intake of micronutrients among children according to study groups and age groups (continued)

| **Micronutrients** | **Case Group (n=30)** | | |  | **Control Group (n=30)** | | |  |  |  |  |
| --- | --- | --- | --- | --- | --- | --- | --- | --- | --- | --- | --- |
|  | **10-13 years** | **14-18 years** | **Total** |  | **10-13 years** | **14-18 years** | **Total** |  |  |  |  |
|  | **X ̄ ± SD**  **Median**  **(Max-Min)** | **X ̄ ± SD**  **Median**  **(Max-Min)** | **X ̄ ± SD**  **Median**  **(Max-Min)** | **p*^c^** | **X ̄ ± SD**  **Median**  **(Max-Min)** | **X ̄ ± SD**  **Median**  **(Max-Min)** | **X ̄ ± SD**  **Median**  **(Max-Min)** | **p*^c^** | **P^c^** | **P1^c^** | **P2^c^** |
| Vitamin B12 (μg) | 7.04±2.06  7.4  (4.0-11.6) | 5.97±3.29  5.3  (1.4-13.7) | 6.50±2.75  6.2  (1.4-13.7) | 0.125 | 4.03±1.95  4.0  (1.4-9.2) | 5.21±3.23  4.0  (1.5-10.7) | 4.50±2.55  4.0  (1.4-10.7) | 0.498 | **0.005** | **<0.001** | 0.421 |
| Folic acid (μg) | 252.32±86.20  221.9  (146.0-466.4) | 280.86±98.98  277.1  (166.6-512.0) | 266.59±92.34  246.1  (146.0-512.0) | 0.340 | 256.47±114.57  231.1  (75.9-442.8) | 233.71±71.32  233.3  (123.0-364.7) | 247.37±98.76  233.3  (75.9-442.8) | 0.703 | 0.483 | 0.986 | 0.283 |
| Calcium (mg) | 926.80±345.12  962.5  (349.1-1478.2) | 843.42±270.08  728.2  (496.7-1237.0) | 885.11±307.43  791.4  (349.1-1478.2) | 0.576 | 759.84±297.77  693.8  (272.2-1454.2) | 812.76±221.20  858.3  (297.1-1151.3) | 781.01±266.89  742.2  (272.2-1454.2) | 0.397 | 0.220 | 0.148 | 0.922 |
| Iron (mg) | 10.89±2.51  11.6  (6.2-14.6) | 11.29±3.02  11.8  (7.3-17.3) | 11.09±2.74  11.7  (6.2-17.3) | 0.868 | 9.20±2.93  9.4  (4.7-16.7) | 8.09±2.30  8.2  (4.3-11.9) | 8.76±2.71  8.6  (4.3-16.7) | 0.253 | **0.002** | 0.065 | **<0.001** |
| Zinc (mg) | 13.45±2.63  13.3  (8.2-18.0) | 12.12±4.40  11.0  (5.9-21.8) | 12.79±3.62  13.2  (5.9-21.8) | 0.263 | 8.70±3.06  8.9  (4.4-16.4) | 9.25±3.54  8.6  (4.7-15.7) | 8.92±3.22  8.9  (4.4-16.4) | 0.687 | **<0.001** | **<0.001** | 0.079 |
| Magnesium (mg) | 276.17±57.32  279.4  (171.3-375.4) | 290.26±83.26  282.3  (194.0-467.0) | 283.21±70.60  281.7  (171.3-467.0) | 0.787 | 261.65±98.84  253.2  (123.1-502.1) | 213.76±57.03  200.8  (149.8-314.9) | 242.49±86.78  223.2  (123.1-502.1) | 0.150 | **0.027** | 0.426 | **0.013** |
| Sodium (mg) | 4637.91±1166.48  4864.8  (2470.7-6714.3) | 4447.03±1493.85  4491.8  (2396.2-8073.7) | 4542.47±1320.46  4678.3  (2396.2-8073.7) | 0.694 | 2205.91±643.73  2232.6  (1169.7-3550.8) | 2810.92±918.11  2511.8  (1956.9-4621.0) | 2447.91±808.41  2299.0  (1169.7-4621.0) | 0.099 | **<0.001** | **<0.001** | **0.002** |
| Potassium (mg) | 2670.51±570.87  2805.1  (1649.8-3892.3) | 2709.67±590.08  2808.7  (1927.2-3754.3) | 2690.09±570.80  2806.9  (1649.8-3892.3) | 0.820 | 2444.59±849.77  2490,5  (1052.7-3882.7) | 2245.74±587.74  2117,8  (1434.5-3490.7) | 2365.05±751.10  2273.6  (1052.7-3882.7) | 0.553 | 0.069 | 0.386 | 0.071 |
| Phosphorus (mg) | 1366.87±276.72  1386.7  (879.2-1710.8) | 1256.77±298.30  1285.0  (742.7-1678.6) | 1311.82±288.20  1360.9  (742.7-1710.8) | 0.272 | 1051.01±363.04  953.6  (544.2-1982.2) | 1013.35±273.15  966.2  (712.4-1493.6) | 1035.95±325.44  966.2  (544.2-1982.2) | 0.933 | **0.001** | **0.010** | **0.038** |

p*: Difference between in-group age groups, P: (total) difference between groups, P1: difference between case-control 10-13 years, P2: difference between case-control 14-18 years,

^c^ Mann-Whitney U test
