# Supplementary figures and images for: Beyond metabolism: nutrition, sleep, and psychological wellbeing in children with insulin resistance—a case-control study
Source: Front Nutr. 2026 Mar 30;13:1781309. doi: 10.3389/fnut.2026.1781309 (PMC13071045; doi:10.3389/fnut.2026.1781309)

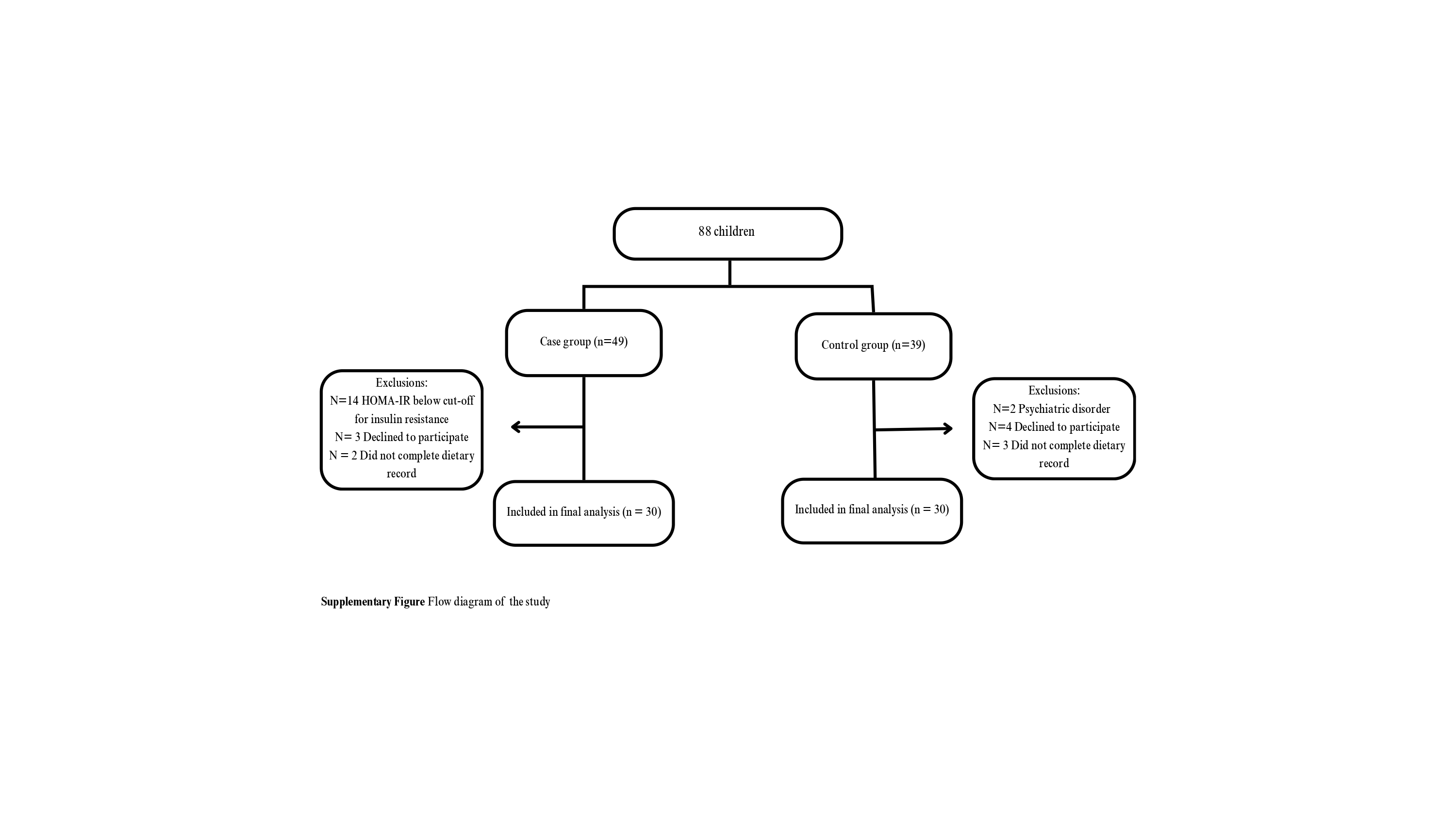

Supplement: Supplementary file 2 [file Image_1.jpeg]
